# Impacts of chronic kidney disease and albuminuria on associations between coronary heart disease and its traditional risk factors in type 2 diabetic patients – the Hong Kong diabetes registry

**DOI:** 10.1186/1475-2840-6-37

**Published:** 2007-12-02

**Authors:** Xilin Yang, Ronald C Ma, Wing-Yee So, Gary T Ko, Alice P Kong, Christopher W Lam, Chun-Shun Ho, Clive S Cockram, Vivian C Wong, Peter C Tong, Juliana C Chan

**Affiliations:** 1 Department of Medicine and Therapeutics, The Chinese University of Hong Kong, Hong Kong SAR, China; 2 Hong Kong Institute of Diabetes and Obesity, The Chinese University of Hong Kong, Hong Kong SAR, China; 3 Li Ka Shing Institute of Health Sciences, The Chinese University of Hong Kong, Hong Kong SAR, China; 4 Department of Chemical Pathology, The Chinese University of Hong Kong, Hong Kong SAR, China; 5 Hospital Authority, Hong Kong SAR, China

## Abstract

**Background:**

Glycated haemoglobin (HbA_1c_), blood pressure and body mass index (BMI) are risk factors for albuminuria, the latter in turn can lead to hyperlipidaemia. We used novel statistical analyses to examine how albuminuria and chronic kidney disease (CKD) may influence the effects of other risk factors on coronary heart disease (CHD).

**Methods:**

A prospective cohort of 7067 Chinese type 2 diabetic patients without history of CHD enrolled since 1995 were censored on July 30^th^, 2005. Cox proportional hazard regression with restricted cubic spline was used to auto-select predictors. Hazard ratio plots were used to examine the risk of CHD. Based on these plots, non-linear risk factors were categorised and the categorised variables were refitted into various Cox models in a stepwise manner to confirm the findings.

**Results:**

Age, male gender, duration of diabetes, spot urinary albumin: creatinine ratio, estimated glomerular filtration rate, total cholesterol (TC), high density lipoprotein cholesterol (HDL-C) and current smoking status were risk factors of CHD. Linear association between TC and CHD was observed only in patients with albuminuria. Although in general, increased HDL-C was associated with decreased risk of CHD, full-range HDL-C was associated with CHD in an A-shaped manner with a zenith at 1.1 mmol/L. Albuminuria and CKD were the main contributors for the paradoxically positive association between HDL-C and CHD for HDL-C values less than 1.1 mmol/L.

**Conclusion:**

In type 2 diabetes, albuminuria plays a linking role between conventional risk factors and CHD. The onset of CKD changes risk associations between lipids and CHD.

## Background

Coronary heart disease (CHD) is one of the leading causes of premature death [1]. Patients with type 2 diabetes have a 2–4 fold increased risk of CHD compared to those without [2]. The United Kingdom Prospective Diabetes Study (UKPDS) has identified hypertension, hyperglycaemia, high low-density-lipoprotein cholesterol (LDL-C), low high-density-lipoprotein cholesterol (HDL-C) and smoking status as major risk factors of CHD [3].

Recent studies have confirmed that albuminuria is another strong risk factor for cardiovascular disease [4-7]. This association holds true for albuminuria which occurs early in life [8]. Glycated haemoglobin (HbA_1c_), blood pressure (BP), HDL-C, smoking and body mass index (BMI) are promoters of albuminuria [9]. The latter has been shown to increase the likelihood of high TC and LDL-C levels in a graded fashion [10]. While this relationship may partly explain the increased risk of CHD in patients with CKD [11], in patients with end-stage renal disease (ESRD), most studies point to low HDL-C but not high LDL-C as risk factors for cardiovascular diseases (CVD) [12].

Although there is some evidence suggesting possible linear risk relationships between HbA_1c_, BP, LDL-C, HDL-C and CHD [3], the linearity of these associations have never been rigorously examined. In this study, we used a non-linear approach to examine the possible impacts of albuminuria and CKD on conventional risk factors and new onset of CHD in a large prospective cohort of Chinese Type 2 diabetic patients.

## Methods

### Subjects

The Prince of Wales Hospital is a regional hospital which serves a population of 1.2 million in Hong Kong. The Hong Kong Diabetes Registry was established in 1995 and enrols 30–50 ambulatory diabetic patients each week. The referral sources included general practitioners, community and specialty clinics and patients discharged from hospitals. Enrolled patients with hospital admissions within 6–8 weeks prior to assessment accounted for less than 10% of all referrals. The 4-hour assessment of complications and risk factors was performed on an outpatient basis, modified from the European DIABCARE protocol [13]. Once a diabetic subject had undergone the comprehensive assessment, he/she was considered to have entered this study cohort and would be followed up till death. The study was approved by the Clinical Research Ethics Committee, Chinese University of Hong Kong. The study complied with the Declaration of Helsinki and written informed consent was obtained from all patients.

Clinical endpoints including discharge diagnoses of hospital admissions and mortality were censored on 30^th ^July 2005. Details of hospital admissions were retrieved from the Hong Kong Hospital Authority Central Computer System which records admissions to all public hospitals in Hong Kong (accounting for 95% hospital beds in Hong Kong). These databases were matched by a unique identification number, the Hong Kong Identity Card number, which is compulsory for all residents in Hong Kong.

Hospital discharge summaries as coded by the International Classification of Diseases, Ninth Revision (ICD-9), were used to identify first CHD. CHD was defined as (1) nonfatal myocardial infarction (code 410), (2) nonfatal ischemic heart disease (code 411–414) and (3) death due to CHD (not including death due to heart failure). Follow-up time was calculated as the period from enrolment to the first CHD event, death or 30^th ^July 2005, whichever came first.

From 1995 to 2005, 7920 diabetic patients were enrolled in the Registry. Among them, 332 with Type 1 diabetes defined as acute presentation with diabetic ketoacidosis, heavy ketonuria (>3+) or continuous requirement of insulin within 1 year of diagnosis [14], and 5 with uncertain type 1 diabetes status, were excluded from the analysis. Forty-nine were excluded due to non-Chinese or unknown nationality. Four hundred and sixty-seven patients were further excluded for having a past history of CHD (including heart failure) at enrolment. A total of 7067 Chinese type 2 diabetic patients without history of CHD and heart failure at baseline were included in this analysis.

### Clinical measurements

Details of assessment methods, definitions and laboratory assays have been previously described [15,16]. On the day of assessment, patients attended the centre after at least 8 hours of fasting and underwent anthropometric measurements and laboratory investigations. We used the Modification of Diet in Renal Disease (MDRD) re-calibrated for Chinese [17] to estimate GFR expressed in ml/min per 1.73 m^2^:

eGFR = 186 × [SCR × 0.011]^-1.154 ^× [age]^-0.203 ^× [0.742 if female] × 1.233

where SCR is serum creatinine expressed as μmol/l (originally in mg/dL, now converted to μmol/l) and 1.233 is the coefficient for Chinese. Peripheral arterial disease (PAD) was defined by the absence of foot pulses on palpation, confirmed by Doppler ultrasound examination of the ankle:brachial ratio <0.90 or treatment for PAD. Chronic kidney disease was defined as eGFR <60 ml/min per 1.73 m^2^. Normoalbuminuria was defined as ACR <2.5 mg/mmol in male and <3.5 mg/mmol in female and microalbuminuria, between 2.5 mg/mmol (male) or 3.5 mg/mmol (female) and 25 mg/mmol, macroalbuminuria, ≥25–150 mg/mmol. Due to its high risk nature, ACR ≥150 mg/mmol was considered as a separate group (see results).

### Statistical analyses

The Statistical Analysis System (SAS, Release 9.10) was used to perform the statistical analysis (SAS Institute Inc., Cary, USA). In order to detect any thresholds, Restricted Cubic Spline (RCS) with 4 knots (i.e. 1 term decomposed into 3 terms: x, x_1 _and x_2_) [18] and Cox proportional hazard regression with the stepwise algorithm (p < 0.05 for entry and stay) were used to obtain a group of significant predictors of CHD. The method on how to use RCS in Cox proportionality has been described in detail by Harrell [18]. The detailed algorithm on how to use a stepwise algorithm in spline Cox regression models has been described elsewhere [19].

Candidate variables at enrolment selected by the spline Cox model included systolic/diastolic blood pressure (BP), HbA_1c_, BMI, waist circumference, blood haemoglobin (Hb), white blood cells (WBC) count, HDL-C, LDL-C, triglyceride (TG), total cholesterol and drug usage (Table 1). After several auto-selection cycles [19], spot urine ACR and eGFR were also included in the final model.

**Table 1:** Baseline clinical and biochemical characteristics of 7067 Chinese Type 2 diabetic patients with no past history of coronary heart disease (CHD)

	Development of CHD before the censoring date				
		
	No (n = 6716)		Yes (n = 351)		
		
	median or %	IQR	median or %	IQR	P value
Follow-up time (years)	5.58	2.97–7.89	2.77	1.48–5.28	<0.001
Male	45.0%	/	51.9%	/	0.012†
Smoking status:					
Current smoker	20.1%	/	29.1%	/	0.057†
Ex smoker	13.2%	/	18.2%	/	
Age (years)	56	46–67	64	54–70	<0.001‡
Sex adjusted waist circumference (cm)*	55.9	0.16	61.9	0.72	<0.001‡
Body mass index (kg/m^2^)	24.7	22.4–27.3	24.6	22.7–26.7	0.748‡
Known duration of diabetes (year)	5	1–10	9	4–14	<0.001‡
Systolic blood pressure (mmHg)	134	121–148	141	130–155	<0.001‡
Diastolic blood pressure (mmHg)	76	69–83	78	70–85	<0.001‡
Peripheral arterial disease	5.4%	/	13.7%	/	<0.001†
Retinopathy	25.6%	/	42.5%	/	<0.001†
Sensory neuropathy	25.5%	/	32.2%	/	0.005†
Glycated haemoglobin (%)	7.3	6.4–8.6	7.6	6.6–9.3	<0.001‡
Blood haemoglobin (g/L)	13.8	12.8–14.9	13.6	12.4–14.7	0.019‡
Spot urinary ACR (mg/mmol)	1.88	0.75–9.81	7.65	1.61–72.3	<0.001‡
Increased albuminuria (ACR ≥3.5 mg/mmol in female & ≥2.5 mg/mmol.in male)	41.2%	/	64.9%	/	<0.001‡
eGFR (ml/min/1.73 m^2^)	105.8	84.9–127.5	89.8	66.0–112.9	<0.001‡
eGFR <60 ml/min/1.73 m^2^	9.3%	/	18.5%	/	<0.001
Low-density lipoprotein cholesterol (mmol/l)	3.10	2.50–3.80	3.52	2.9–4.2	<0.001‡
High-density lipoprotein cholesterol (mmol/l)	1.25	1.05–1.50	1.14	0.99–1.37	<0.001‡
Triglyceride (mmol/L)	1.37	0.96–2.06	1.55	1.11–2.18	<0.001‡
Total cholesterol (mmol/L)	5.20	4.50–5.90	5.54	4.85–6.30	<0.001†
Baseline use of drugs:					
Oral anti-diabetic drugs	60.9%	/	59.0%	/	0.448†
Anti-hypertensive drugs	33.2%	/	43.0%	/	<0.001†
Insulin	17.0%	/	25.9%	/	<0.001†
Lipid lowering drugs	12.3%	/	13.7%	/	0.450†
ACEI or ARB	20.0%	/	25.1%	/	0.022†

IQR, inter-quartile range; eGFR, estimated glomerular filtration rate; ACEI, Angiotensin-converting enzyme inhibitors; ARB, angiotensin II receptor blockers; ACR, albumin:creatinie ratio; *, derived from analysis of covariance; †, derived from Chi-square text; ‡, derived from Wilcoxon two-sample test.

In exploratory analysis, we calculated hazard ratio (HR) changes over full-ranges of baseline risk factors before and after adjustment for eGFR and ACR, in order to observe the impacts of albuminuria and CKD on these risk associations. Hazard ratio between two points of variable X_i _can be estimated by exp (y_2 _- y_1_), where y_1 _and y_2 _are the corresponding RCS function values of two X_i _points. In this study, the 25^th ^or 75^th ^percentile (for near linear relationship) or zenith points (for non linear relationship) of baseline variables were chosen as the reference point (y_1_) to estimate HR of other points of baseline variable X_i _(y_2_). Here, y (y_1 _and y_2_) was the RCS function value of X_i_, which was calculated by the formula: the spline function value of X_i _= βx+βx_1_+βx_2_, where β, β_1 _and, β_2 _were estimated by applying x, x_1 _and x_2 _as covariates in Cox models.

We then categorised significant continuous risk factors identified in the HR plots and used Cox regression analysis to confirm the findings in the risk curve analysis. Proportionals hazards assumption and functional form were checked using Supremum test [20], which is implemented using ASSESS statement in the SAS procedure PROC PHREG. A *p*-value of <0.05 for two-sided tests was considered to be statistically significant.

## Results

### Study population and predicting models

At enrolment, the median age of the cohort was 57 years (interquartile range [IQR]: 46–67 years) with a median disease duration of 5 (IQR: 1–11) years. During a median follow-up period of 5.40 (IQR: 2.87–7.81) years, 351 (4.97%) patients developed incident CHD giving an incidence rate of CHD of 9.28 (95% CI: 8.31–10.24) per 1000 person-years. During the follow-up period, 681 (9.64%) patients died. Of these, 47 deaths were due to fatal CHD (included in the 351 events, CHD as the principal diagnosis). Patients who developed CHD were older, had longer duration of diabetes, more unfavourable lipid profile (LDL-C, HDL-C and TG), worse renal function, higher HbA_1c_, urinary ACR and WBC, lower Hb and were more likely to be treated with insulin and antihypertensive drugs at baseline that those who did not (Table 1).

The spline Cox model selected sex, smoking status (current smoker/ex-smoker), use of angiotensin-converting enzyme inhibitors (ACEI)/angiotensin II receptor blockers (ARB) and spline terms of age, duration of diabetes, TC, HDL-C, blood Hb and insulin use at enrolment (Model 1). Blood Hb (p = 0.1709) and insulin use (p = 0.1710) were no longer significant after further inclusion of spline term of eGFR, while all other variables remained significant. Further adjusting for spline term of ACR (p for ACR = 0.0041) did not change the significance of other variables in the model.

### Risk factors of coronary heart disease

**Figure 1:**
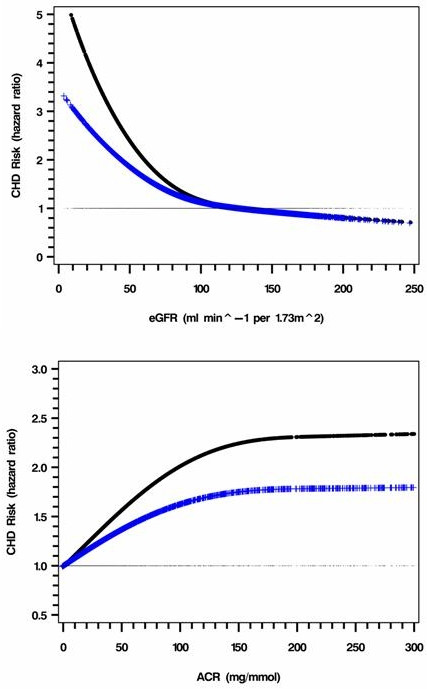
**Full range risk associations between CHD and eGFR/ACR**. a. Black: adjusted for model 1 variables (p < 0.05); Blue: further adjusted for ACR (p < 0.05). Model one variables include age, sex, and smoking status (current/ex), total cholesterol, HDL-C, Hb, eGFR and use of ACEI/ARB as well as use of insulin at enrolment. The hazard ratio was calculated using the 25^th ^percentiles, 75^th ^percentiles as the reference level. b. Black: adjusted for model 1 variables (p < 0.05); Blue: further adjusted for eGFR (p < 0.05).

Estimated GFR was negatively associated with incident CHD (Figure 1 and Table 2). The HR for CHD started to rise at the trough value of 100 ml/min per 1.73 m^2^, and rapidly from 60 ml/min per 1.73 m^2 ^downwards. The HR of ACR for CHD increased rapidly from 0 to 150 mg/mmol before reaching a plateau. Similar trends with ACR was observed in patients with normo, micro and macroalbuminuria and those with ACR ≥150 mg/mmol (p < 0.05 for trend) (Figure 1 and Table 2).

**Table 2:** Hazard ratios of significant baseline risk factors for coronary heart disease in type 2 diabetic patients (Model 1) before and after inclusion of eGFR and ACR into the model.

	Model 1†		Model 1 plus eGFR‡		Model 1 plus eGFR & ACR¶	
	
Vaseline variables	Hazard ratio (95% CI)	*p-*value	Hazard ratio (95% CI)	*p-*value	Hazard ratio (95% CI)	*p-*value
**Age (year):**						
<45 vs. 45–59	0.48 (0.28–0.81)	0.006	0.51 (0.30–0.86)	0.012	0.50 (0.29–0.84)	0.010
≥60 vs. 45–59	1.75 (1.31–2.33)	<0.001	1.56 (1.16–2.09)	0.003	1.53 (1.14–2.06)	0.005
**Female sex**	0.64 (0.47–0.88)	0.006	0.66 (0.48–0.91)	0.010	0.69 (0.50–0.95)	0.023
**Smoking status:**						
Ex-smoking vs. never	1.21 (0.84–1.76)	0.311	1.18 (0.81–1.71)	0.384	1.13 (0.78–1.64)	0.5202
Current vs. never	1.64 (1.15–2.33)	0.007	1.63 (1.15–2.33)	0.007	1.58 (1.11–2.26)	0.011
**Duration of diabetes (year):**						
<5 vs. ≥13	0.43 (0.30–0.61)	<0.001	0.45 (0.31–0.64)	<0.001	0.49 (0.34–0.71)	<0.001
5–12 vs. ≥13	0.71 (0.53–0.97)	0.028	0.74 (0.54–1.00)	0.048	0.78 (0.58–1.06)	0.118
**Blood haemoglobin (g/dL):**						
<12.5 vs. ≥12.5 g/dL	1.58 (1.16–2.15)	0.004	1.35 (0.98–1.87)	0.067	1.22 (0.88–1.69)	0.238
**HDL-C (mmol/L):**						
<0.80 vs. 0.80–1.39	0.41 (0.18–0.93)	0.033	0.39 (0.17–0.88)	0.024	0.40 (0.18–0.90)	0.027
≥1.40 vs. 0.80–1.39	0.52 (0.38–0.70)	<0.001	0.54 (0.39–0.73)	<0.001	0.56 (0.41–0.76)	<0.001
**Total cholesterol (mmol/L)**	1.22 (1.11–1.36)	<0.001	1.20 (1.08–1.32)	<0.001	1.12 (1.00–1.25)	0.045
**Use of ACEI or ARB**	1.72 (1.29–2.31)	<0.001	1.59 (1.18–2.13)	0.002	1.44 (1.06–1.94)	0.018
**Use of insulin**	1.34 (0.99–1.81)	0.056	1.26 (0.93–1.70)	0.136	1.15 (0.85–1.56)	0.372
**eGFR (ml/min per 1.73 m**^2^**)**:						
<60 vs. ≥90	/	/	1.90 (1.27–2.84)	0.002	1.37 (0.89–2.10)	0.152
60–89.9 vs. ≥90	/	/	1.51 (1.12–2.06)	0.008	1.36 (0.99–1.85)	0.055
**Urinary ACR (mg/mmol)ξ**						
Microalbuminuria	/	/	/	**/**	1.34 (0.97–1.85)	0.075
Macroalbuminuria	/	/	/	**/**	1.76 (1.19–2.58)	0.043
ACR ≥150 mg/mmol	/	/	/	**/**	2.64 (1.69–4.12)	<0.001

eGFR, estimated glomerular filtration rate; ACR, albumin: creatinine ratio; HDL-C, high-density lipoprotein cholesterol; ACEI, Angiotensin-converting enzyme inhibitors; ARB, angiotensin II receptor blockers ξ, Microalbuminuria is defined as ACR≥2.5 mg/mmol in male and ≥3.5 mg/mmol in female but <25 mg/mmol in both genders; macroalbuminuria here is defined as ≥25 mg/mmol but <150 mg/mmol in both genders. †, -2log likelihood without covariates = 4108.15 and with covariates = 3909.48; ‡, -2log likelihood with covariates = 3897.88; ¶, -2log likelihood with covariates = 3879.21.

**Figure 2:**
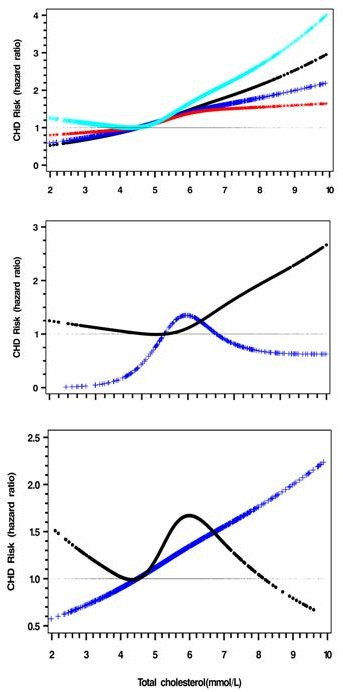
**Full range risk associations between total cholesterol and CHD before and after adjustment for eGFR and ACR**. a. Black: derived from model 1 (p < 0.05); Blue: further adjusted for eGFR (p < 0.05); Red: further adjusted for eGFR and ACR (p < 0.05); Cyan: limited to eGFR ≥60 ml/min per 1.73 m^2^in model 1 (p < 0.05). Model one variables include age, sex, and smoking status (current/exit), total cholesterol, HDL-C, Hb, eGFR and use of ACEI/ARB as well as use of insulin at enrolment. b. Black: adjusted curve in patients with eGFR ≥60 ml/min per 1.73 m^2 ^(p < 0.05); Blue: adjusted curve in patients with eGFR <60 ml/min per 1.73 m^2 ^(p < 0.05). c. Black: adjusted curve in patients without albuminuria (p = 0.080); Blue: adjusted curve in patients with albuminuria (p < 0.05).

There was near linear association between TC and CHD risk, which was attenuated by adjustment for eGFR and ACR (Figure 2a). Exclusion of patients with CKD led to a higher HR for those with high TC >5.0 mmol/L. In patients without CKD, the HR of TC for CHD started to increase linearly from 5.0 mmol/L upwards. In patients with CKD, the shape of the risk curve was changed to one of "A-shaped" with a peak HR at 5.0 mmol/L (Figure 2b). In patients with normoalbuminuria, there was no significant association between TC and CHD (Figure 2c). Conversely, in patients with albuminuria, there was a linear relationship between TC and CHD risk.

**Figure 3:**
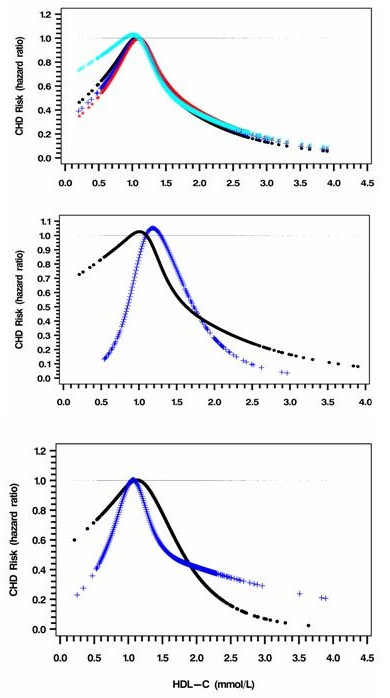
**Full range risk associations between HDL-C and CHD before and after adjustment for eGFR and ACR**. a. Black: derived from model 1 (p < 0.05); Blue: further adjusted for eGFR (p < 0.05); Red: further adjusted for eGFR and ACR (p < 0.05); Cyan: limited to eGFR ≥60 ml/min per 1.73 m^2^in model 1 (p < 0.05). Model one variables include age, sex, and smoking status (current/ex), total cholesterol, HDL-C, Hb, eGFR and use of ACEI/ARB as well as use of insulin at enrolment. The hazard ratio was calculated using the zenith as the reference level. b. Black: adjusted curve in patients with eGFR ≥60 ml/min per 1.73 m^2 ^(p < 0.05); Blue: adjusted curve in patients with eGFR <60 ml/min per 1.73 m^2 ^(p < 0.05). c. Black: adjusted curve in patients without albuminuria (p < 0.05); Blue: adjusted curve in patients with albuminuria (p < 0.05).

HDL-C was associated with CHD in an A-shaped manner with a zenith at 1.1 mmol/L and a long tail on the right (Figure 3a). Both HDL-C ≥1.40 mmol/L and HDL-C <0.80 mmol/L were associated with reduced risk of CHD (Table 3b), which remained significant after adjusting for eGFR and ACR. The gradient of the HR curve accelerated more rapidly from very low level of HDL-C up to 1.1 mmol/L in the albuminuric group than in the non-albuminuric group. After excluding patients with CKD (n = 690), the negative risk association between CHD and HDL-C was significant for HDL-C ≥1.40 mmol/L (p < 0.001) but not for HDL-C level <0.80 mmol/L, p = 0.127).

**Figure 4:**
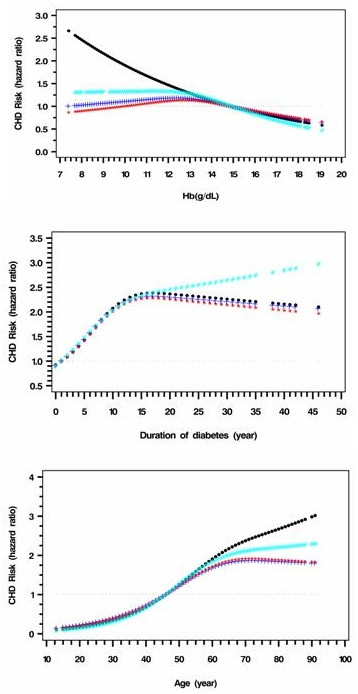
**Full range risk associations between CHD and Hb/duration of diabetes/age before and after adjustment for eGFR and ACR**. a. Black: adjusted for model 1 variables (p < 0.05); Blue: further adjusted for eGFR (p: NS). Red: further adjusted for eGFR and ACR (p: NS); Cyan: limited to eGFR ≥60 ml/min per 1.73 m^2 ^in model 1 (p < 0.05). Model one variables include age, sex, and smoking status (current/ex), total cholesterol, HDL-C, Hb, eGFR and use of ACEI/ARB as well as use of insulin at enrolment. The hazard ratio was calculated using the 25^th ^percentiles, 75^th ^percentiles as the reference level. b. Black: adjusted for model 1 variables (p < 0.05); Blue: further adjusted for eGFR (p < 0.05). Red: further adjusted for eGFR and ACR (p < 0.05); Cyan: limited to eGFR ≥60 ml/min per 1.73 m^2^in model 1 (p < 0.05). c. Black: adjusted for model 1 variables (p < 0.05); Blue: further adjusted for eGFR (p < 0.05). Red: further adjusted for eGFR and ACR (p < 0.05); Cyan: limited to eGFR ≥60 ml/min per 1.73 m^2 ^in model 1 (p < 0.05)

Blood Hb was associated with CHD risk in a linear manner (Figure 4). Excluding patients with CKD changed the shape of the HR curve with a shoulder value at 12.5 g/dL. Adjusting for eGFR also rendered the HR non significant for Hb <12.5 g/dL versus. ≥12.5 g/dL (Table 2). Risk of CHD increased with disease duration during the first 13 years, which then maintained at a high level (Figure 4 and Table 2). Old age, male gender, current smokers, use of ACEI/ARB and use of insulin were also associated with higher risk of CHD (Figure 4 and Table 2).

## Discussions

Our study re-affirms previous observations that age, male gender, tobacco intake, long disease duration, high TC, low HDL-C, high ACR, and low eGFR were independent risk factors of CHD using conventional Cox regression analysis. The novelty of our analysis lies in its ability to demonstrate the powerful effects of albuminuria and CKD on modifying these risk relationships as evidenced by changes in the HR plots of these risk factors. In particular, the risk association between blood Hb and CHD was entirely explained by eGFR and ACR while albuminuria and CKD had profound effects on the CHD risk association with HDL-C and TC.

### Lipid parameters

The UKPDS reported graded increase in CHD risk with LDL-C in type 2 diabetes [3]. In our model, instead of LDL-C, TC was selected as a risk factor of CHD. More detailed analysis revealed complex interplay between lipid parameters, albuminuria, CKD and CHD risk in this large prospective cohort of type 2 diabetic patients with a broad range of renal function and albuminuria.

While we observed a near linear relationship between TC and CHD, once ACR was fitted into the risk curve, this relationship was present only in patients with albuminuria. These findings concur with that from a large scale cross-sectional study in USA (n = 17,702) which showed risk association between albuminuria and hypercholesterolemia (TC and LDL-C) in a graded fashion [10]. On the other hand, the linear association between CHD and TC at ≥5.0 mmol/L was mainly observed in patients without CKD. Using the non-linear approach, we further demonstrated that high HDL-C was associated with low CHD risk when HDL-C was ≥1.1 mmol/L. However, for levels lower than 1.1 mmol/L, the presence of albuminuria and, in particular, CKD markedly changed the shape of the risk curve to an 'A shaped' with a paradoxically positive association between CHD risk and HDL-C level, giving rise to a zenith value of 1.10 mmol/L. These non-linear relationships were further confirmed by conventional Cox regression analysis. Using 0.80–1.39 mmol/L as the referent, HDL-C <0.80 mmol/L and HDL-C ≥1.4 mmol/L were both associated with reduced risk of CHD.

The nature of this positive association between HDL-C and CHD for HDL-C values less than 1.1 mmol/L, observed mainly in patients with CKD or albuminuria requires further elucidation. However, in light of the potent anti-inflammatory and anti-oxidant properties of HDL-C particles [21,22], we postulate that these associations may be due to changes in the metabolic milieu associated with CKD and severe albuminuria [23,24].

Against these thought-provoking findings, it is noteworthy that in the recent 4D study, treatment with atorvastatin failed to reduce CHD risks in patients with ESRD [25]. These findings are not unexpected given the lack of association between LDL-C and CHD in patients with ESRD in epidemiological studies as well as the non-association between TC and CHD risk in our patients with CKD [12]. Furthermore, two recent clinical trials failed to confirm the hypothesis that increasing HDL-C can reduce the progression of coronary atherosclerosis [26,27]. Again, our findings regarding the powerful effects of albuminuria and CKD on altering the pattern of risk association between HDL-C and CHD highlight the complexity of interrelationships between energy metabolism and organ function.

### Blood haemoglobin, renal impairment and albuminuria

Our group and others have reported the risk association of CHD with low eGFR [11,28]. In our current analysis, adjustment for ACR greatly attenuated the association between eGFR and CHD risk, suggesting that the risk association was in part mediated by albuminuria, a marker of endothelial dysfunction. In this cohort, we detected a sharp and linear association between CHD risk and ACR starting from 0 to 150 mg/mmol. This observation therefore concord with findings by Gerstein *et al *showing that any degree of albuminuria is a risk factor for cardiovascular disease [29].

There is strong evidence showing that low blood Hb is a strong predictor for CHD [30,31]. In our analysis, the risk association between CHD risk and blood Hb was rendered non significant after adjustment for eGFR and exclusion of patients with CKD. These findings suggest that blood Hb may merely serve as a surrogate marker for CKD and thus may explain the negative results of two recent clinical trials which failed to confirm the beneficial effects of correction of anaemia using erythropoietin therapy on cardiovascular endpoints in patients with ESRD [32,33].

### Other CHD risk factors

In patients with diabetes less than 13 years, there was linear relationship between CHD risk and disease duration. In patients with disease more than 13 years, the statistical significance of disease duration disappeared. This may be confounded by the strong relationship between disease duration and albuminuria and that between albuminuria and hypercholesterolaemia [9,10].

Age is a well-known risk factor of CHD [3]. However, our study suggests that this age-associated CHD risk was in part mediated by loss of renal function after the age of 55 years. In agreement with the UKPDS [3], we also found a risk-protecting effect of female gender on CHD. Smoking is a well-known risk factor of CHD which is also independently associated with CHD in our cohort [3].

Although there is strong epidemiological evidence supporting the risk association between CHD and glycemic control [3,34], the UKPDS failed to confirm the benefits of improving glycemia on CHD rates in an interventional setting [35]. In our cohort, HbA_1c _was a significant predictor for CHD with a HR of 1.07 for every 1% increase in HbA_1c _(p = 0.0136) after controlling for age, sex, SBP and smoking status. However, this significance was rendered non significant once ACR, TC, HDL-C, or disease duration were adjusted for. Other studies have shown that improvement in glycemic control reduced albuminuria and hypercholesterolaemia [35-37]. Taken together, with the possible causal effect of albuminuria on hypercholesterolaemia [10], our findings suggest that the effect of HbA_1c _on CHD risk is likely to be mediated through risk factors such as albuminuria and lipids.

Blood pressure is a strong risk factor for CHD in type 2 diabetes [3]. In our analysis, the age and sex adjusted hazard ratio of SBP for CHD was 1.23 (95% CI: 1.07–1.18) per 10 mmHg (p < 0.001). However, after adjusting for the spline term of ACR, the significance of SBP did not persist (p = 0.172). Removal of the use of ACEI/ARB in the spline Cox model (without ACR and eGFR), the spline term of SBP was significant (p = 0.007). These findings suggest that low BP and use of ACEI/ARB were associated with reduced risk of CHD, largely mediated by albuminuria.

Similar to the findings from the UKPDS [3], BMI and waist circumference were not selected as risk factors of CHD in the model. The age and sex adjusted HRs were also not significant (p = 0.494 for BMI and 0.182 for waist circumference). Although BMI has been implicated in albuminuria [9], the association between BMI and CHD may be confounded by other mediators such as dyslipidemia and inflammation. Besides, the prognostic significance of BMI in the presence of co-morbidities such as diabetes may become paradoxically reversed [38].

## Limitations

This prospective cohort consists of a heterogeneous cohort of type 2 diabetic patients with a wide range of disease duration and risk factors. Although this heterogeneity and the use of single baseline values may theoretically reduce the precision of these risk estimations, this draw back was partly compensated by the relatively large number of clinical events, detailed phenotyping at baseline and long period of observation. Overall, results generated from both conventional and non-linear approaches are robust and consistent which have generated alternative hypotheses which are biologically plausible. Further clinical and experimental studies are required to confirm these findings.

## Conclusions and Implications

Using a large prospective database and relatively novel and robust statistical methods, we have found a strong linear association between TC and CHD only in patients with albuminuria. Adjusting for eGFR and albuminuria attenuated the associations between lipid, Hb, BP, duration of diabetes and CHD, suggesting that albuminuria plays a linking role between these risk factors and CHD. The onset of CKD further changes risk associations between lipids (such as TC and HDL-C) and CHD. Recently, several major randomised clinical trials have yielded negative results regarding the effects of correcting anemia and reducing LDL-C on cardiovascular outcomes in patients with ESRD as well as that of raising HDL-C on reducing progression of atherosclerosis.

Based on these observations, we infer the following pathways to CHD in type 2 diabetes: 1). Hyperglycaemia and hypertension lead to albuminuria, a marker of endothelial and renal damage; 2). Albuminuria leads to hyperlipidaemia which further increases the risk of CHD; and 3). Albuminuria, both as a surrogate for multiple risk factors and causal factors, leads to deterioration of renal function and 5). Reduced renal function further changes the pattern of risk association between HDL-C and CHD, i.e., the predictive value of very low HDL-C (<0.8 mmol/L) no longer holds when CKD has developed.

Understanding the complex relationships among risk factors of CHD in type 2 diabetes is an important step towards further reducing CHD risk in type 2 diabetes. For example, reducing albuminuria might further control hyperlipidaemia and enhance the benefits of controlling traditional risk factors such HbA_1c_, BP and LDL-C. Our data also suggest that retarding rate of deterioration of renal function and correcting anaemia may have important cardioprotective effects. However, these hypotheses will need to be confirmed by both experimental and interventional studies.

## Abbreviations

ACEI: Angiotensin-converting enzyme inhibitor;

ACR: Albumin: creatinine ratio;

ARB: Angiotensin II receptor blockers;

BMI: Body mass index;

CHD: Coronary heart disease;

CKD: Chronic kidney disease;

eGFR: Estimated glomerular filtration rate;

ESRD: End-stage renal disease;

Hb: Haemoglobin;

HbA_1c_:Glycated haemoglobin;

HR: Hazard ratio;

HDL-C: High density lipoprotein cholesterol;

ICD-9: International Classification of Diseases, Ninth Revision;

LDL-C: Low-density-lipoprotein cholesterol;

MDRD: Modification of Diet in Renal Disease;

PAD: Peripheral arterial disease;

RCS: Restricted Cubic Spline;

UKPDS: United Kingdom Prospective Diabetes Study;

SCR: Serum creatinine;

SBP/DBP/BP: Systolic/diastolic blood pressure;

TC: Total cholesterol;

TG: Triglyceride;

WBC: White blood cell.

## Competing interests

The author(s) declare that they have no competing interests.

## Authors' contributions

XLY performed the statistical analysis and drafted the manuscript. JC, RM, WS, GK, AK, CSC, PT and GC were involved in study design, coordination and data acquisition. VW, CL and CSH facilitated retrieval of laboratory data and clinical outcomes. All authors have read and approved the final manuscript.
